# TgMORN2, a MORN Family Protein Involved in the Regulation of Endoplasmic Reticulum Stress in *Toxoplasma gondii*

**DOI:** 10.3390/ijms241210228

**Published:** 2023-06-16

**Authors:** Jinxuan Li, Qianqian Xiao, Qianqian Tan, Junpeng Chen, Lingyu Sun, Xiao Chen, Ziyu Chu, Hongxia Wu, Zhenzhao Zhang, Hongmei Li, Xiaomin Zhao, Xiao Zhang

**Affiliations:** 1 Department of Preventive Veterinary Medicine, College of Veterinary Medicine, Shandong Agricultural University, Tai’an 271018, China; a18853850827@163.com (J.L.); xiaoqq970419@163.com (Q.X.); tanqianqian2022@163.com (Q.T.); chenjunpeng1223@163.com (J.C.); sunlingyu0813@163.com (L.S.); chexpp@163.com (X.C.); chuziyu1999@163.com (Z.C.); zhangzhenzhao2022@163.com (Z.Z.); lihm@sdau.edu.cn (H.L.);; 2 Shandong Provincial Key Laboratory of Animal Biotechnology and Disease Control and Prevention, Shandong Agricultural University, Tai’an 271018, China; 3 Shandong Provincial Engineering Technology Research Center of Animal Disease Control and Prevention, Shandong Agricultural University, Tai’an 271018, China

**Keywords:** *Toxoplasma gondii*, TgMORN2, endoplasmic reticulum stress

## Abstract

MORN proteins play a key role in the cytoskeletal structure of eukaryotes and are essential for the close arrangement of the endoplasmic reticulum and plasma membrane. A gene with nine MORN motifs (TGGT1_292120, named Tg*MORN2*) was identified in the *Toxoplasma gondii* genome; it was presumed to belong to the MORN protein family and to have the function of forming the cytoskeleton, which affects the survival of *T. gondii*. However, the genetic deletion of *MORN2* did not noticeably affect parasite growth and virulence. Using adjacent protein labeling techniques, we identified a network of TgMORN2 interactions, which mainly included endoplasmic reticulum stress (ER stress)-related proteins. In exploring these data, we found that the pathogenicity of the KO-Tg*MORN2* strain was significantly reduced in the case of tunicamycin-induced ER stress. Reticulon TgRTN (TGGT1_226430) and tubulin β-Tubulin were identified as interaction proteins of TgMORN2. Collectively, TgMORN2 plays a role in ER stress, which lays a foundation for further research on the function of the MORN protein in *T. gondii*.

## 1. Introduction

*T. gondii* is a protozoan parasite of *Phylum Apicomplexa* that infects most warm-blooded animals, such as humans and cats [1]. The outcome of toxoplasmosis in the host varies. Immunocompetent individuals infected with *T. gondii* are often asymptomatic or develop mild symptoms. However, it may cause serious illness and death in immunodeficient individuals and in the developing fetus of pregnant women [2]. The pathogenesis of *T. gondii* is due to its rapid replication cycle, which causes destructive tissue lesions. The replication cycle takes only about six to seven hours to complete [3,4,5] and is dependent on the proper formation of the cytoskeleton [6,7,8,9,10,11,12,13]. Due to the importance of cytoskeleton development, the components of this structure have become attractive potential therapeutic targets and have become the basis for an increasing number of research areas [14].

In 2000, Takeshima et al. first proposed the existence of MORN domain in mammalian junctophilin-1 (JPH1) [15]. Subsequently, MORN structural domain proteins were discovered in animals and plants, such as the MORN4 of *Drosophila* [16], the amyotrophic lateral sclerosis protein ALS2 [17], and the radial spoke protein 44 (RSP44) [18]. The characterized plant proteins that harbor MORN motifs are phosphatidylinositol monophosphate kinases, chloroplasts 3 (ARC3) protein [19], and the *Brassica rapa* MORN motif protein (BrMORN) [20]. MORN motifs play critical roles in several proteins with roles in the organization of membranous and cytoskeletal structures [21]. Examples of these are the junctophilins, which are critical for the tight appositions of endoplasmic reticulum and plasma membrane in excitable cells [15]. Loss or mutation of these proteins prevents junctional complex assembly and leads to neuronal dysfunction. In addition, MORN repeats act as protein–protein or protein–phospholipid binding domains. For example, plant phosphatidylinositol kinases (PIPKs) contain MORN motifs whose repeats can bind to phospholipids and localize to the plasma membrane or other biomembranes [21].

MORN is highly conserved among apicomplexan parasites. MORN1, MORN2, and MORN3 have been identified in *T. gondii* [22]. Previous studies have reported a distinct sub-cellular localization of TgMORN1 in *T. gondii*, suggesting that TgMORN1 acts as a scaffolding protein and organizing protein complex with a critical role in mitosis and cytokinesis [10,23,24]. TgMORN1 deficiency results in defective organelle partitioning and basal complex assembly [10,11]. This ultimately leads to double-headed parasites. TgMORN2 contains nine MORN conserved motifs and nine plasma membrane-binding structural domains [23], whose function is currently unknown.

In the current study, we found that TgMORN2 and TgMORN1 were functionally different. The Δ*MORN2* tunicamycin-induced endoplasmic reticulum stress was more sensitive. Moreover, using Biotin labeling technology with molecular validation demonstrated that TgMORN2 interacted with *T. gondii* reticulon (TgRTN) and β-Tubulin.

## 2. Results

### 2.1. Generation and Verification of TgMORN2 Gene Editing Strains

**Figure 1:**
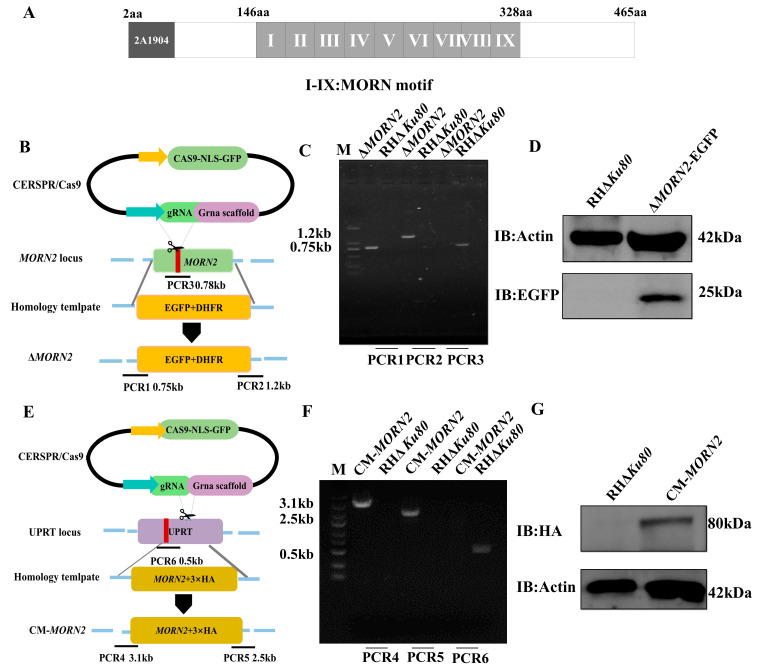
The Δ*MORN2* and CM-*MORN2* strains were successfully constructed. (**A**) TgMORN2 (TGGT1_292120) domain analysis (9 MORN conserved motifs in grey text box). (**B**) Schematic illustrating CRISPR-CAS9-mediated gene knockout by insertion of EGFP and pyrimethamine-resistant DHFR-Ts into the coding sequence of *MORN2*. (**C**) PCR1, PCR2, and PCR3 analyses confirming the knockout of the target gene. (**D**) Western blot identified activation of the GFP tag by the promoter of MORN2. (**E**) Schematic diagram of the CRISPR/CAS9-mediated MORN2-HA insertion at the UPRT locus. PCR4 and PCR5 detected the correct insertion sites, and PCR6 detected the UPRT endogenous locus. (**F**) Diagnostic PCR demonstrating homologous integration and gene disruption in MORN2-HA strain compared with the parental line RHΔ*Ku80*. (**G**) Immunoblot assays checking the expression of MORN2-HA fusions in transgenic parasites.

TgMORN2 is mainly formed by nine MORN repeats (Figure 1A). MORN2 has recently been further localized to the apical end of the parasite [22], and it was found to be associated with the cytosolic leaflet of the plasma membrane fraction in the hyperplexed localization of organelle proteins by isotope tagging (hyper LOPIT) studies [25]. To further understand its function, a knockout mutant of TgMORN2 was constructed in the RHΔ*Ku80* [26] strain by double homologous recombination using CRISPR/CAS9 technology (Figure 1B). PCR1 and PCR2 determined the correct integration of enhanced green fluorescent protein (EGFP) and dihydrofolate reductase (DHFR) into the genome, and PCR3 was used to determine the complete knockout of the *MORN2* gene (Figure 1C). Western blotting results indicated that the EGFP protein was expressed in the knockout strain at a size of 25 kDa, while it was not detected in the RHΔ*Ku80* strain. This indicates that the EGFP tag is activated by the TgMORN2 promoter and that TgMORN2 is correctly replaced by the EGFP tag (Figure 1D). The TgMORN2-hemagglutinin (HA) construct was co-transfected with the uracil phosphoribosyltransferase (UPRT)-targeting CRISPR plasmid into Δ*MORN2* to generate the complementary strain CM-*MORN2* (Figure 1E), with clones being generated and identified by PCR (Figure 1F). By probing with anti-HA, MORN2-HA fusion was efficiently expressed (Figure 1G). 

### 2.2. The TgMORN2 Is Dispensable for Parasite Growth

**Figure 2:**
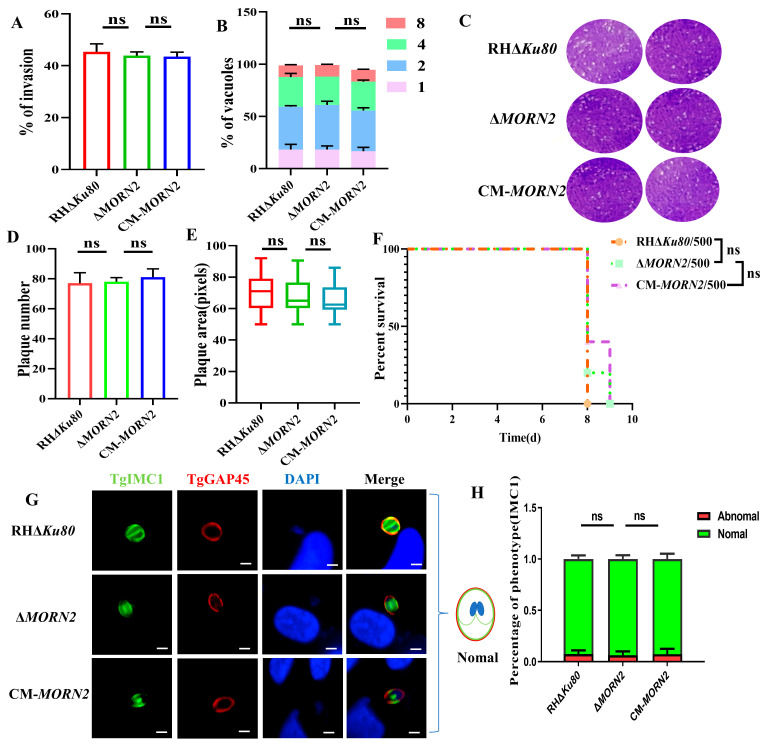
Absence of *MORN2* did not affect parasite invasion and proliferation. (**A**) Differential invasion efficiency of RHΔ*Ku80*, Δ*MORN2*, and CM-*MORN2* strains; *n* = 3, a representative experiment from two independent assays; ns: not significant. (**B**) Intracellular replication assay for evaluating in vitro proliferation of RHΔ*Ku80*, Δ*MORN2*, and CM-*MORN2* strains. Tachyzoites were counted in 100 PVs. Data represent mean ± SEM for two independent experiments. (**C**) Plaque assays used to compare the overall growth capacity of RHΔ*Ku80*, Δ*MORN2*, and CM-*MORN2* strains. (**D**,**E**) The number and area of the plaque assay; ns: not significant. (**F**) Survival curves of BALB/c mice (500 tachyzoites per mouse; *n* = 5 mice per group) infected with RHΔ*Ku80*, Δ*MORN2*, or CM-*MORN2* strains. The mice were divided into three groups. Every mouse was monitored daily until death; ns: not significant. (**G**) Utilization of IFA to observe *T. gondii* division. TgIMC1: inner membrane complex of progeny; TgGAP45: glideosome-associated protein; TgIMC1 protein (green) and TgGAP45 (red) strains body profile. Scale, 2.5 μm; DAPI (nuclear dye): 4’,6-Diamino-2-phenylindole. (**H**) Graph shows the percentages of vacuoles in RHΔ*Ku80*, Δ*MORN2*, and CM-*MORN2* strains. Randomly selected vacuoles (*n* = 100) from two independent experiments were quantified.

The cytoskeleton of *T. gondii* is known to be essential for its growth, and consequently, it is required for virulence [10]. Subsequently, we determined the effect of the *MORN2*’s deletion on parasite growth. An invasion assay, replication assay, and plaque assay showed that the deletion of *MORN2* did not affect the invasion and proliferation of *T. gondii* (Figure 2A–E). A virulence assay in mice showed that *MORN2* was dispensable for *T. gondii* pathogenesis (Figure 2F). The cell division of RHΔ*Ku80*, Δ*MORN2*, and CM-*MORN2* strains was evaluated [27]. The localization of the progeny inner membrane complex TgIMC1 [11] was monitored using the cell maturation marker TgGAP45 [7] as a reference, and the results showed that the Δ*MORN2* strain had normal IMC1 localization, indicating that the Δ*MORN2* strain divided normally (Figure 2G–H). These results suggested that *MORN2* knockout had no effect on the virulence of *T. gondii* and that TgMORN2 may not be associated with cytoskeleton formation. There is a little speculation that other proteins cooperate with TgMORN2 to participate in *T. gondii* life activities.

### 2.3. Identification of the TgMORN2 Interactome

**Figure 3:**
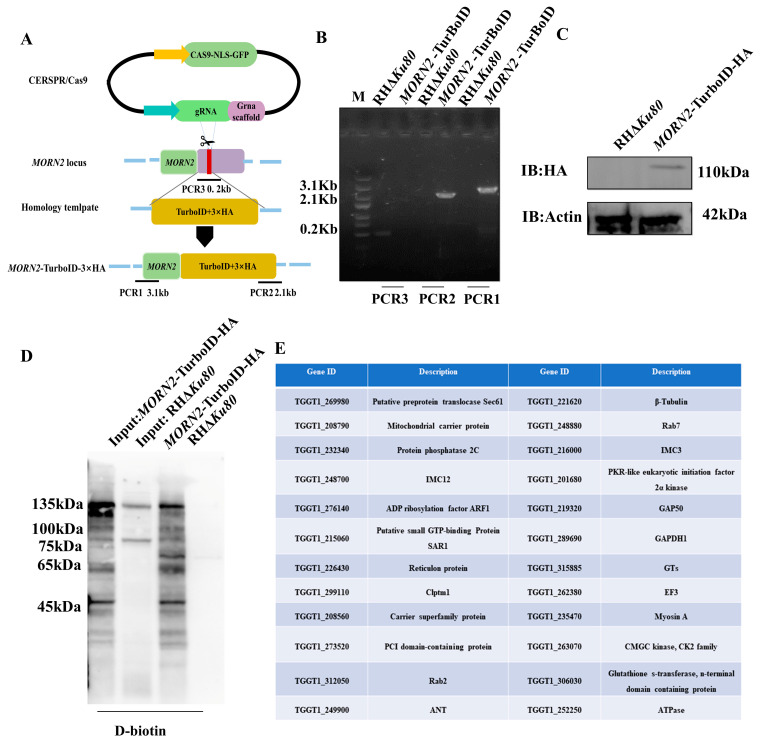
MORN2-TurboID-HA-directed protein biotinylation. (**A**) Schematic diagram of CRISPR/CAS9-mediated insertion of TurboID-HA into the C-terminus of TgMORN2. PCR1 and PCR2 detected the correct insertion sites, and PCR3 detected the *MORN2* endogenous locus. (**B**) Diagnostic PCR demonstrating homologous integration and gene disruption in the Tg*MORN2*-TurboID-HA strain compared to the parental line RHΔKu80. (**C**) Immunoblot assays to check the expression of TgMORN2-TurboID-HA fusions in transgenic parasites. (**D**) Visualization of biotinylated proteins in parasites. Western blot comparing the profile of biotinylated proteins from lysates of RHΔ*Ku80* and Tg*MORN2*-TurboID-HA parasites. Biotinylated proteins were detected by horseradish peroxidase (HRP)-conjugated streptavidin. (**E**) Analysis of mass spectrometry data.

To identify the interacting proteins of TgMORN2, we utilized the BioID system using TgMORN2 as a bait protein. A recombinant RHΔ*Ku80* strain was constructed. Using CRISPR, the TurboID with a hemagglutinin (HA) tag was fused to the C terminus of TgMORN2 (MORN2-TurboID-HA) (Figure 3A). The TurboID-HA tag integrated into the C-terminal of TgMORN2 was identified by PCR (Figure 3B). Western blotting using anti-HA antibodies showed that the recombinant strain correctly expressed TgMORN2-TurboID-HA and migrated at the expected size (Figure 3C). Biotinylated proteins were enriched from parasite lysates by streptavidin-conjugated beads, which were confirmed by Western blotting (Figure 3D) and analyzed by mass spectrometry for identification. (Figure 3E). After the removal of common contaminants and the control RHΔ*Ku80* proteins, and by analyzing the MORN family based on a synthesis of relevant reports, 24 reliable proteins were detected in the Tg*MORN2*-TurboID-HA parasite samples. They were related to the ER stress, membrane protein, and metabolism in *T. gondii*.

### 2.4. Non-Interaction of TgMORN2 with TgIMC3, TgIMC12, and TgGAP50

**Figure 4:**
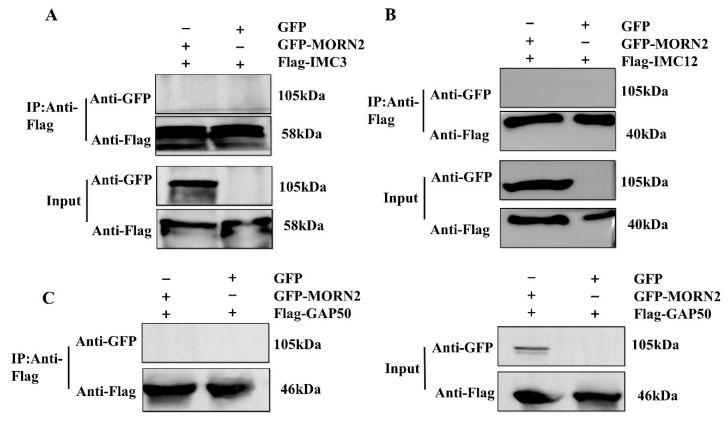
Non-interaction of TgMORN2 with TgIMC3, TgIMC12, and TgGAP50. (**A**) Non-co-precipitation of pEGFP-MORN2 with pCMV-Flag-IMC3 in HEK293T cells. After expression and differentiation, cells were harvested for Co-IP and further analysis by Western blotting using anti-GFP and anti-Flag antibodies. (**B**) Non-co-precipitation of pEGFP-MORN2 with pCMV-Flag-IMC12 in HEK293T cells. The experimental treatment is shown in (**A**). (**C**) Non-co-precipitation of pEGFP-MORN2 with pCMV-Flag-GAP50 in HEK293T cells. The experimental treatment is shown in (**A**).

The membrane proteins TgIMC3 [8], TgIMC12 [8], and TgGAP50 [28] in mass spectrometry, which are also cytoskeleton proteins of *Toxoplasma*, were selected to verify their relationship with TgMORN2. To determine whether TgMORN2 interacted with TgIMC3, co-immunoprecipitation (Co-IP) was performed on HEK293T cells transfected with plasmids expressing the pCMV-Flag-IMC3 and pEGFP-MORN2 proteins. Co-IP was performed using Flag mAb-coated beads, and the eluates were detected using GFP and Flag monoclonal antibody (mAb), respectively. As a result, GFP-MORN2 could not be pulled down by the Flag-IMC3 (Figure 4A), indicating non-interaction with TgMORN2 and TgIMC3. Co-IP was also performed to further verify the interaction between GFP-MORN2 and Flag-IMC12 or Flag-GAP50. The immunoprecipitation results showed that TgMORN2 failed to co-immunoprecipitate with TgIMC12 (Figure 4B) and TgGAP50 (Figure 4C). This further indicates that TgMORN2′s function is different from that of TgMORN1 [10].

### 2.5. Interaction of TgMORN2 with Endoplasmic Reticulum Stress-Related Protein TgRTN

**Figure 5:**
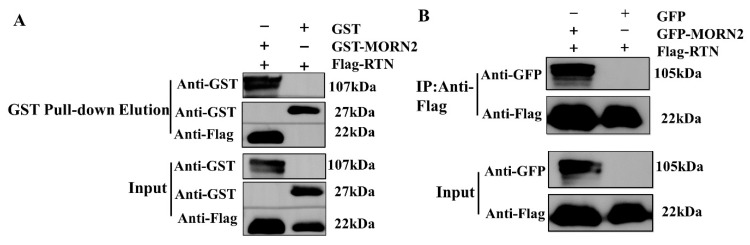
TgMORN2 interacts with TgRTN. (**A**) GST pull-down assays. GST or GST-MORN2 fusion protein generated by *Escherichia coli* BL21 was purified by glutathione agarose resin, followed by incubation of the resin with Flag-RTN protein expressed in HEK293T cells. After washing, the bound proteins were analyzed by Western blotting with anti-Flag antibodies. (**B**) Co-precipitation of pEGFP-MORN2 with pCMV-Flag-RTN in HEK293T cells. After expression and differentiation, cells were harvested for Co-IP and further analyzed by Western blotting using anti-GFP and anti-Flag antibodies to reveal that pEGFP-MORN2 interacts with pCMV-Flag-RTN. The input was used as the positive control.

In addition, *T. gondii* reticulon (TgRTN) was selected in the mass spectrum for verification. RTN4 has been shown to be involved in ER stress and inflammation in murine myocytes [29]. The reticulon protein plays an important role in bending and shaping the endoplasmic reticulum (ER) membrane [30]. TgRTN was selected based on mass spectrometry analysis results to validate its interaction with TgMORN2 and to assess the potential implications of TgMORN2 in ER stress. We conducted the GST pull-down assay to verify this interaction. Western blotting using an anti-Flag antibody was performed to examine the precipitates. As shown in Figure 5A, Flag-RTN was precipitated by GST-MORN2 and not by GST alone. To further verify the interaction between the TgMORN2 and TgRTN, the constructed plasmids pEGFP-MORN2 and pCMV-Flag-RTN were co-transfected into HEK293T cells. The results showed that Flag-RTN co-precipitates with GFP-MORN2 (Figure 5B). Therefore, the interaction between TgMORN2 and TgRTN was confirmed by Co-IP.

### 2.6. TgMORN2 and TgRTN Interact with β-Tubulin

**Figure 6:**
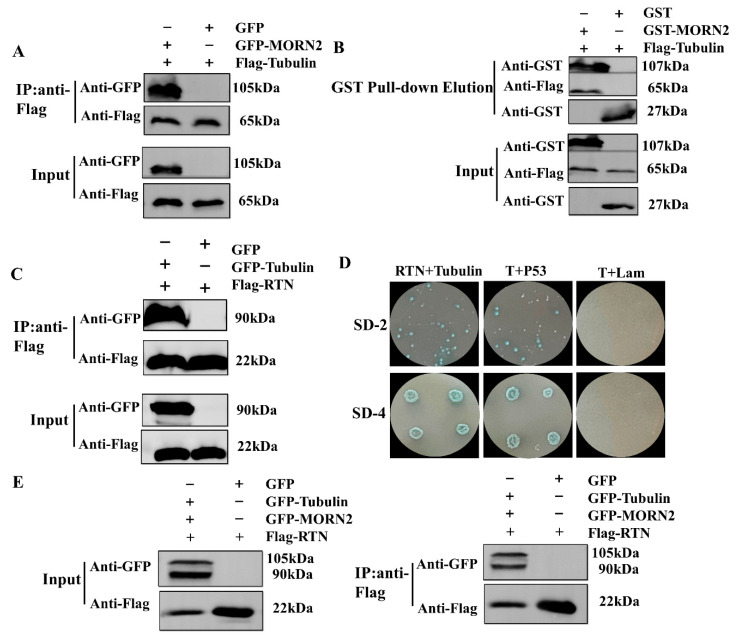
β-Tubulin interacts with TgMORN2 and TgRTN. (**A**) HEK293T cells were transfected with pEGFP-MORN2 and pCMV-Flag-β-Tubulin. The immunoprecipitation interaction assay between pEGFP-MORN2 and pCMV-Flag-β-Tubulin was performed by Western blotting analysis. The input was used as the positive control. (**B**) TgMORN2 binding to β-Tubulin in vitro, using the GST pull-down assay. The control GST and GST-MORN2 fusion protein were purified with GST-agarose, after being mixed with the cell lysates containing β-Tubulin-Flag protein, and the interaction was detected by Western blotting. (**C**) Co-immunoprecipitation of TgRTN with β-Tubulin. PEGFP-β-Tubulin was co-expressed with pCMV-Flag-RTN in HEK293T cells. The isolated protein was analyzed by immunoblotting with anti-Flag antibodies to detect TgRTN and anti-GFP antibodies to detect β-Tubulin. The input was used as the positive control. (**D**) Analysis of the interaction between β-Tubulin and RTN by Y2H system. TgRTN, p53, and LaminC (Lam) were cloned into the pGBKT7 vector, while β-Tubulin and LargeT (T) were cloned into the pGADT7 vector, respectively. T+p53 and T+Lam were used as positive and negative controls for the yeast two-hybrid system, respectively. (**E**) Conducting Co-IP to detect the interaction between TgMORN2, β-Tubulin, and TgRTN in HEK293T cells. The input was used as the positive control.

It has previously been reported that reticulum proteins can interact with tubulin in nerve cells [26]. Therefore, β-Tubulin was selected based on the mass spectrometry results to further verify its interaction with MORN2. To confirm whether TgMORN2 binds to β-Tubulin, Co-IP assays were performed in HEK293T cells transfected with pEGFP-MORN2 and pCMV-Flag-β-Tubulin. The results showed that GFP-MORN2 was detected in the sediment (Figure 6A), suggesting that β-Tubulin and MORN2 interact in vivo. In addition, a GST pull-down assay demonstrated that TgMORN2 interacted directly with the β-Tubulin in vitro (Figure 6B). This further confirmed that TgMORN2 interacts with β-Tubulin. As TgMORN2 interacts with TgRTN and β-Tubulin, the relationship between TgRTN and β-Tubulin needs to be explored. To examine this interaction, Co-IP assays was performed. The pEGFP-β-Tubulin and pCMV-Flag-RTN plasmids were co-expressed in HEK293T cells. As shown in Figure 6C, a specific signal for GFP-β-Tubulin was clearly observed in the Flag-RTN immunoprecipitation. Subsequent yeast two-hybrid (Y2H) experiments revealed that co-transformation of pGBKT7-RTN and pGADT7-β-Tubulin into Y2HGold yeast cells resulted in growth on SD/-Leu/-Trp/-Ade/-His + X-α-gal (SD-4) plates and blue color development (Figure 6D). Additionally, Co-IP analysis confirmed the interaction among the TgRTN, TgMORN2, and β-Tubulin proteins. Flag-RTN was detected using anti-Flag antibodies, and GFP-tagged TgMORN2 and β-Tubulin could be detected by anti-GFP immunoblotting (Figure 6E). Previous studies suggested an intrinsic link between ER stress responses and the microtubule network [31]. Therefore, we speculate that TgMORN2 may be related to the endoplasmic reticulum stress of *T. gondii*.

### 2.7. More Pronounced Induction of Endoplasmic Reticulum Stress by Tunicamycin in the ΔMORN2 Strain

**Figure 7:**
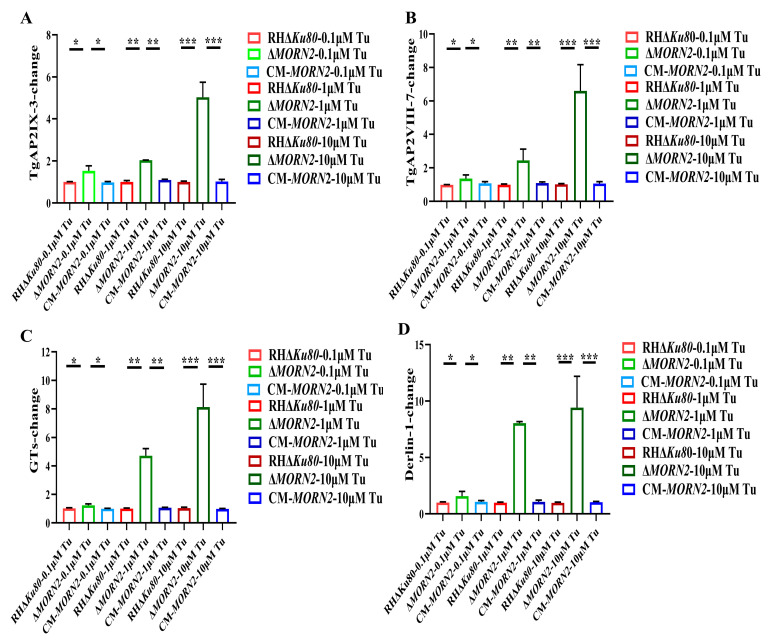
More pronounced induction of ER stress by tunicamycin in the ΔMorn2 strain. (**A**–**D**) Measurement of upregulation of TgAP2IX-3, TgAP2VIII-7, GTs, and Derlin-1 by qPCR. Histograms represent the transcriptional changes of the four genes in RHΔ*Ku80*, Δ*MORN2*, and CM-*MORN2* strains as stimulated by Tu. Values shown are means ± SEM from three independent experiments (*n* = 3), each with three replicates. *, *p* < 0.05; **, *p* < 0.01; ***, *p* < 0.001, Student’s *t*-test.

Tunicamycin (Tu) was initially identified as a natural antibiotic, and by inhibiting protein glycosylation to become a canonical antibiotic, Tu can potently trigger ER stress [30,32,33,34]. *T. gondii* Apetela-2IX-3 (TgAP2IX-3), *T. gondii* Apetela-2VIII-7 (TgAP2VIII-7), Glycosyltransferases (GTs), and Derlin-1 have previously been associated with ER stress in *T. gondii*, and their upregulation after Tu stimulation has been described [33]. To confirm the conjecture that TgMORN2 may be involved in ER stress, different concentrations of tunicamycin were used to stimulate the parasites for 1 h. In comparison with the RHΔ*Ku80* group, the levels of TgAP2IX-3 [35,36], TgAP2VIII-7 [35,36], Glycosyltransferases [37,38], and Derlin-1 [37,38] (ER stress correlative factors in *T. gondii*) [33] in the Δ*MORN2* strain were significantly increased (Figure 7A–D). In short, these data suggest that TgMORN2 plays a key role in the ER stress of *T. gondii*.

### 2.8. Morn2 Deletion Leading to Intolerance of T. gondii to Endoplasmic Reticulum Stress

**Figure 8:**
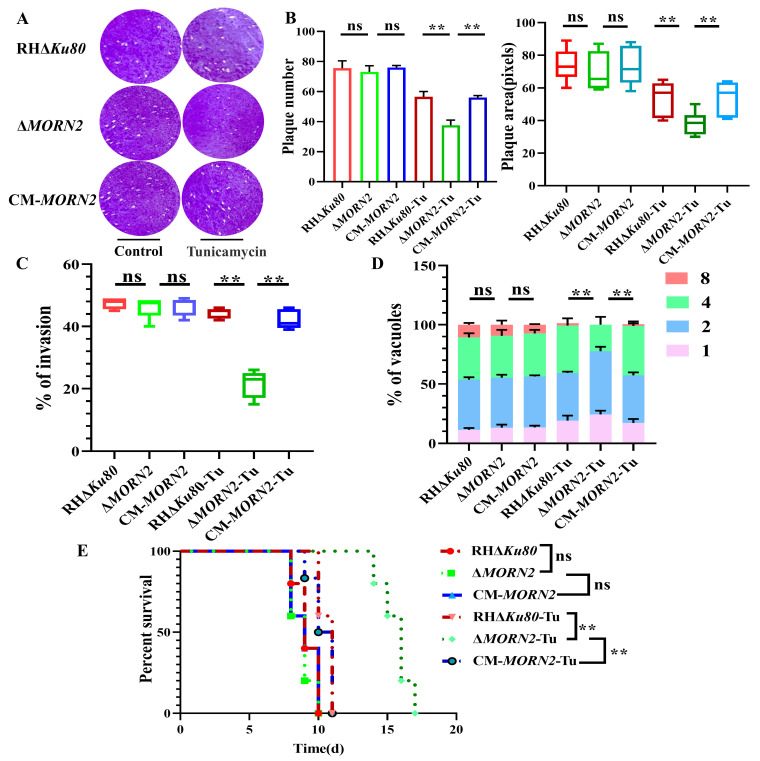
Loss of *MORN2* causes intolerance of *T. gondii* to ER stress. (**A**) Plaque assays were used to compare the overall growth ability of the RHΔ*Ku80*, Δ*MORN2* and CM-*MORN2* strains. (**B**) The number and area of the plaque assay; ns: not significant; **, *p* < 0.01. (**C**) Different invasion efficiencies of Tu-stimulated RHΔ*Ku80*, Δ*MORN2*, and CM-*MORN2* strains; ns: not significant; **, *p* < 0.01. Graph represents mean ± SEM for two independent experiments. (**D**) Intracellular replication assay used to evaluate the proliferation of RHΔ*Ku80*, Δ*MORN2*, and CM-*MORN2* strains in vitro after stimulation with Tu; ns: not significant; **, *p* < 0.01. Two-way ANOVA mean ± SEM for three independent experiments. (**E**) Survival curve of mice infected with the designated strains. RHΔ*Ku80*, CM-*MORN2*, and Δ*MORN2* strains stimulated with 10 μM were infected with BALB/c mice by intraperitoneal injection. Each group was composed of five mice, and a mouse was challenged with 500 tachyzoites. Every mouse was monitored daily until death; ns: not significant; **, *p* < 0.01, Gehan–Breslow–Wilcoxon tests.

After stimulation with 10 μM Tu [33], the intracellular replication of the Δ*MORN2* strain was significantly reduced (Figure 8C–D). For the plaque assay, the Δ*MORN2* strains treated with tunicamycin also formed significantly smaller and fewer plaques compared to the RHΔ*Ku80* and CM-*MORN2* strains (Figure 8A–B). Similarly, the pathogenicity of the knockout strain was attenuated in tunicamycin-treated mice (Figure 8E). The above evidence suggests that the deletion of TgMORN2 leads to the altered survival of *T. gondii* in the context of ER stress.

## 3. Discussion

The experimental results suggested that the deletion of *MORN2* did not affect the invasion and proliferation of *T. gondii* in vitro. We developed BioID technique to rapidly identify and determine the function of TgMORN2. The results showed that TgRTN and β-Tubulin were the interacting proteins of TgMORN2. Under ER stress, the total loss of Tg*MORN2* resulted in a significant decrease in parasite invasion, intracellular proliferation, and pathogenicity in mice, demonstrating the involvement of MORN2 in the ER stress response of *T. gondii*.

Unlike TgMORN2, TgMORN1 is most prominently localized to the basal complex of the IMC [23,24,39]. The absence of TgMORN1 leads to incomplete cytokinesis, with particularly incomplete abscission and budding, and to the formation of multi-headed parasites [11]. However, the absence of *MORN2* had no significant effect on the division and proliferation of *T. gondii*. In addition, the overexpression of TgMORN1 severely perturbed the parasite nuclear division and cytokinesis [23]. Further evidence suggests that TgMORN1 interacts with the cytoskeleton of the parasite [14]. In contrast, we verified that TgMORN2 indeed fails to interact with IMC. The above evidence shows that TgMORN2 is another MORN family protein and is different from TgMORN1.

ER should stimulate the ability of saturated ER proteins to fold under many physiological and pathological conditions, leading to the accumulation of false-folding proteins [33]. When the ER is stressed, eukaryotic cells upregulate the expression of stress-responsive genes through a mechanism called the unfolded protein response (UPR). The UPR is more like a series of ‘emergency’ measures from the cell for maintaining homeostasis, such as slowing down protein translation and triggering autophagy [40]. For example, preferentially translated AP2 factors serve as the parasite counterparts of bZIP transcription factors and coordinate the gene expression of UPR in other species [41]. Following acute exposure to tunicamycin, many induced genes play integral roles in protein processing (folding, degradation, and vesicle transport), lipid biosynthesis, and oxidative stress. The main findings include the upregulation of GTs and Derlin-1 [33,38], which is essential for the degradation of misfolded ER proteins. In this study, TgAP2IX-3, TgAP2VIII-7, GTs, and Derlin-1 were significantly upregulated in the tunicamycin-stimulated Δ*MORN2* strain. However, further investigations are needed to elucidate the role of TgMORN2 in the UPR and its involvement in endoplasmic reticulum stress.

Microtubules are known to regulate ER homeostasis, with ER dynamics being closely linked to the dynamics of microtubules [42]. This is particularly important during ER stress, as ER expansion is one of the relief mechanisms. In nerve cells, Nogo-B interacts with tubulin and its localization is consistent [43]. As reported in previous studies [44], disruption of the β-Tubulin: CCT-β complex apparently triggers both protein degradation systems, thus forcing target cells towards ER stress-associated apoptosis. Therefore, the hypothesis that β-Tubulin also interacts with TgRTN was derived, and subsequent experiments supported this speculation. However, it is not clear whether the simultaneous deletion of TgMORN2 and TgRTN affects the survival of *T. gondii*. Thus, further research is called for.

With the exception of the few proteins that have already been validated in this study, the mass spectrometry data also showed the presence of ANT and Rabs. TgANT is important for the maintenance of mitochondrial morphology [2,45]. In addition, its depletion significantly inhibited the proliferative capacity of *T. gondii* in vitro and its pathogenicity in mice. The absence of ANT has been shown to cause a sharp increase in the unfolded or misfolded proteins, resulting in endoplasmic reticulum stress [45]. Recently, the small GTPases Rab10 [46] and Rab18 [47] have been reported to control ER shape by regulating ER dynamics and fusion. Depletion of Rab7a leads to basal ER stress [48], which in turn leads to ER membrane expansion. Previous studies on junctophilins have shown that MORN repeats can provide attachment to the ER cytoplasmic face by interacting with phospholipids. Based on our research, we can speculate that ANT/Rabs may be involved in the endoplasmic reticulum stress together with TgMORN2. However, this assumption needs further validation.

In short, endoplasmic reticulum stress in *T. gondii* is a complex process in many ways. The possible involvement of TgMORN2 in the expansion of the endoplasmic reticulum or the formation of phagosomes during endoplasmic reticulum stress in *T. gondii* remains to be studied.

## 4. Materials and Methods

### 4.1. Parasite Strain, Cells and Mice

Type I strain RHΔ*Ku80* was used to construct the transgenic strain, which was maintained in DF-1 cells (a spontaneously immortalized chicken embryo fibroblast cell line) or Vero cells (African green monkey kidney cells) at 37 °C and 5% CO_2_ using cell culture flasks (NEST Biotechnology, Wuxi, China). Four-week-old female BALB/c mice were purchased from Jinan Pengyue Experimental Animal Breeding Co., Ltd. and kept under standard conditions in accordance with the regulations specified by the Administration of Affairs Concerning Experimental Animals.

### 4.2. Construction of Transgenic Strains

*MORN2* deletions in RH*ΔKu80* strains were performed by the CRISPR/Cas9 system, which utilized homologous recombination to replace the target gene, and the associated method was performed as described previously [49]. To construct the complementary strain, RHΔ*Ku80 MORN2*::3xHA and sgUPRT CRISPR plasmids were coelectroporated and integrated into the UPRT locus to replace the original TgUPRT. The monoclonal strain was screened by immunofluorescence using mAb HA. The positive clones were further analyzed by PCR for correct integration at the TgUPRT locus, and the TgMORN2-HA expression was analyzed by Western blotting.

### 4.3. Immunoblotting and Immunofluorescence Assays

The immunofluorescence assays (IFAs) were performed as described previously [49]. Briefly, tachyzoite-infected DF-1 cells were fixed with 4% paraformaldehyde. After three washes with PBS, the cell membranes were permeabilized and the samples were blocked by incubation with 0.25% Triton X-100/PBS and 3% bovine serum albumin for 30 min at room temperature. The primary antibodies used were mouse anti-HA (1:500; Sigma Aldrich, St. Louis, MO, USA), rabbit anti-GAP45 (1:250), rabbit anti-SAG1 (1:150), and rabbit anti-IMC1 (1:200). TgActin, TgSAG1, and TgIMC1 were prepared by our laboratory. The location of the primary antibody was visualized by using a mixture of FITC-conjugated goat anti-mouse IgG [H+L] (1:75) and Cy3-conjugated goat anti-rabbit IgG [H+L] (1:250). The nuclear material was co-stained with 4′,6-diamidino-2-phenylindole (DAPI).

For the immunoblotting assays, 10^7^ parasites were collected and purified by filtration through a 5 µm filter membrane and lysed with RIPA buffer (Solarbio, Beijing, China). The primary antibodies used were mouse anti-GFP (1:6000; Abways, Shanghai, China), mouse anti-HA (1:8000; Sigma Aldrich, St. Louis, MO, USA), and mouse anti-Actin (1:500). Horseradish peroxidase-conjugated antibodies were used as secondary antibodies (1:5000; CWBIO, Beijing, China).

### 4.4. Invasion Assay

A total of 10^4^ freshly released tachyzoites of different strains were inoculated into confluent DF-1 cells grown on coverslips. After 30 min of parasite invasion, the cells were washed three times with PBS. The extracellular parasites were stained with mouse anti-TgSAG1, and the total parasites were stained with rabbit anti-TgGAP45. Fluorescein isothiocyanate (FITC)-conjugated goat anti-mouse IgG (H+L) and Cy3-conjugated goat anti-rabbit IgG (H+L) were secondary antibodies and were incubated together. The invasion efficiency was calculated by counting the ratio of the number of infected tachyzoites/total host cells in several random fields under a fluorescence microscope [50]. All the strains were tested 3 times independently.

### 4.5. Proliferation Assay 

A total of 10^4^ freshly released tachyzoites of different strains were inoculated into confluent DF-1 cells grown on coverslips. After 2 h of parasite invasion, the cells were washed with PBS and the culture medium was changed. After 24 h, the cells on the coverslips were fixed and subjected to IFA (staining for parasites using anti-GAP45). Tachyzoites in 100 parasitophorous vacuoles (PVs) were counted in several random fields visualized under fluorescence microscopy [51]. 

### 4.6. Plaque Assays 

The plaque assays were performed as described previously [50]. Briefly, 500 freshly released tachyzoites were inoculated into confluent DF-1 cell monolayers grown in 12-well plates. After 7 days of culture, the cells were washed with PBS, then fixed with 4% formaldehyde for 20 min, stained with 2% crystal violet for 10 min, washed with PBS, dried, and imaged. Thirty plaques were measured in each condition.

### 4.7. Virulence Assay in Mice 

Five-week-old female BALB/c mice were infected with 500 tachyzoites by intraperitoneal injection. The survival rate was monitored for 20 days post-infection. The virulence test was repeated three times.

### 4.8. Western Blot Analysis

For the Western blots, the samples resolved by 12% SDS-PAGE were transferred onto polyvinylidene fluoride (PVDF) membranes. Non-specific protein interactions were blocked with 5% skim milk in TBST buffer (TBS buffer containing 0.05% Tween-20) for 1 h at room temperature. The membranes were incubated in a 5% skim milk buffer for 1 h at room temperature with the appropriate primary antibodies. The blotted membranes were washed six times in TBST buffer and then incubated with 1:5000 dilutions of horseradish peroxidase-conjugated secondary antibodies in TBST buffer for 1 h. The blotted membranes were washed six times in TBST buffer. The proteins were visualized using bioluminescence reagents (New Cell and Molecular Biotech, Suzhou, China) in accordance with the manufacturer-specified protocol.

### 4.9. Affinity Purification of Biotinylated Proteins

To screen for interacting proteins of TgMORN2, biotin-adjacent labeling was performed as described previously [49]. Briefly, the TurboID and 3× HA tags were fused to the C-terminal end of the MORN2 coding sequence. Transcription of MORN2-TurboID-3× HA was initiated by the *T. gondii* MORN2 promoter. Purified PCR products of the TurboID-3× HA cassette were co-transfected with the CRISPR/Cas9 plasmid into RHΔ*Ku80* parasites and selected with DHFR after electroporation. Vero cells monolayers infected with the Tg*MORN2*-TurboID-HA strain were cultured in DMEM containing 150 μM D-biotin for 24 h as the experimental group. The RHΔ*Ku80* strain with biotin treatment was used as the control. The biotinylated proteins were analyzed by Western blotting using HRP-labeled streptavidin (Sangon Biotech, Shanghai, China), while the remaining samples were loaded in 12% SDS-PAGE gel and separated for about 2 h. The protein bands of the control and experimental groups were cut and sent to the BGI Genomics (Shenzhen, China) Company for mass spectrometry identification.

The following steps were performed at the BGI Genomics (Shenzhen, China) Company. The identification of protein gel strips was to separate the sample proteins by gel electrophoresis; then, the protein gel strips were obtained at different positions on the film; the peptides were extracted after enzymatic digestion; then, mass spectrometry (MS) was used to obtain the mass spectrum of the proteins in these gel strips, and finally, the protein identification software (Mascot v2.3, Matrix Science, London, UK) was used to identify the proteins in the samples. The whole process started from the conversion of the raw MS data into a peak list; then, matches were searched for in the database. The databases used included the UniProt protein database and the genome annotation-based protein database. The search results were subject to strict filtering and quality control, and possible protein identifications were produced.

### 4.10. Co-Immunoprecipitation

HEK293T cells co-transfected with the corresponding plasmids were lysed in 400 µL of RIPA buffer containing protease inhibitors and immunoprecipitated with protein A+G (Beyotime, Shanghai, China). The experiment was continued according to the manufacturer’s instructions by first adding 1 μL of mouse IgG anti-Flag to the lysate and then adding it to the protein A+G. The bound proteins were eluted, and the samples were subjected to Western blotting. The primary antibodies used were mouse anti-GFP (1:6000; Abways, Shanghai, China) and mouse anti-Flag (1:8000; *Abways*, Shanghai, China). Horseradish peroxidase-conjugated antibodies were used as secondary antibodies (1:5000; CWBIO, Beijing, China). 

### 4.11. Yeast Two-Hybrid (Y2H) Assay

The interaction between TgRTN and β-Tubulin was verified by Y2H assay, as described previously [49]. The full-length cDNA of TgRTN was amplified and cloned into vector pGBKT7 to construct a bait plasmid. The cDNA sequence of β-Tubulin was amplified and cloned into vector pGADT7 to construct the prey plasmid. After being confirmed by sequencing, both prey and bait plasmids were co-transformed into yeast strain Y2HGold. The interaction of each co-transformation combination was verified by growing the co-transformants on minimal −Leu/−Trp (SD-2) medium and −Leu/−Trp/−His/−adenine (SD-4) medium containing 20 mg/mL X-α-gal. Photographs were taken after 5 days of yeast cell growth on −Leu/−Trp (SD-2) medium or −Leu/−Trp/−His/−adenine (SD-4) medium.

### 4.12. Fusion Constructs and GST Pull-down Assays

pCMV-Flag-RTN and pCMV-Flag-β-Tubulin were transfected in HEK293T. The GST-fusion protein of TgMORN2 was recombinantly expressed in *E. coli* BL21. The cells were pelletized and lysed by sonication in protein extraction buffer. After centrifugation at 10,000× *g* for 30 min, the bacterial lysates were coupled to glutathione sepharose beads (Beyotime, Shanghai, China) for 4 h. The beads were washed 3 times with washing buffer and subsequently incubated with HEK293T cell lysates for 4 h. After 12% SDS-PAGE, the proteins were transferred onto PVDF membranes using the Western blot method. The primary antibodies used were rabbit or mouse anti-GST (1:8000; Proteintech, Wuhan, China) and rabbit or mouse anti-Flag (1:8000; Abways, Shanghai, China). Horseradish peroxidase-conjugated antibodies were used as secondary antibodies (1:5000; CWBIO, Beijing, China).

### 4.13. RNA Extraction and qPCR

The tachyzoites of the RH∆*Ku80*, Δ*MORN2*, and CM-*MORN2* strains were stimulated with 0.1 μM, 1 μM, and 10 μM Tu for 1 h. Total RNA was extracted using TRIzol reagent following the manufacturer’s suggestions. The extracted RNA was reverse transcribed into cDNA using the StarScript II Reverse Transcriptase (GenStar Co., Ltd., Beijing, China); then, the target gene expression levels were measured using qPCR with SYBR qPCR Master Mix (*Vazyme*, Nanjing, China) using LightCycler^®^96 (Roche, Basel, Switzerland). TgGADPH was used as a reference gene. The qPCR program included a denaturation step at 94 °C for 30 s, followed by 40 cycles of 94 °C for 5 s and 60 °C for 30 s. The comparative 2^−ΔΔCt^ method was applied to analyze the relative levels of gene expression.

### 4.14. Statistical Analysis

All the data were analyzed in Prism 8 (GraphPad Software, Inc., La Jolla, CA, USA) using Student’s *t*-tests, as indicated in the figure. The statistical data were expressed as the mean value of the standard error of the mean (SEM). All the analyses were performed with a two-tailed Student’s *t*-test, except for the parasite proliferation assay and the mouse virulence assay, which were analyzed with a two-way ANOVA and the Gehan–Breslow–Wilcoxon test. *p* < 0.05 was considered statistically significant. Significance was indicated by asterisks according to the following scale: ns, *p* > 0.05; *, *p* < 0.05; **, *p* < 0.01; ***, *p* < 0.001.

## Data Availability

Data openly available in a public repository.

## Abbreviations

MORN	membrane occupation and recognition nexus protein
ALS	amyotrophic lateral sclerosis protein
ARC	chloroplasts
ANT	putative adenine nucleotide translocator
BrMORN	*Brassica rapa* MORN motif
Co-IP	co-immunoprecipitation
Clptm1	cleft lip and palate transmembrane protein1
DHFR	dihydrofolate reductase
RSP	radial spoke protein
JPH	junctophilin
PIPKs	plant phosphatidylinositol kinases
RTN	reticulon
LOPIT	localization of organelle proteins by isotope tagging
EGFP	enhanced green fluorescent protein
EF3	putative elongation factor Tu
ER	endoplasmic reticulum
GAP45	anchors the gliding machinery
GADPH	glyceraldehyde-3-phosphate dehydrogenase
GTs	putative glycosyltransferase
HA	hemagglutinin
IMC	inner membrane complex
mAb	monoclonal antibody
PVs	parasitophorous vacuoles
Rab	putative small GTPase
SD-2	SD-Leu-Trp
SD-4	SD-Leu-Trp-His-Ade
TgAP2IX-3	*T. gondii* Apetela-2IX-3
TgAP2VIII-7	*T. gondii* Apetela-2 VIII-7
Tu	tunicamycin
UPRT	phosphoribosyltransferase
UPR	unfolded protein response
